# Feasibility and Health Effects of a 15-Week Combined Exercise Programme for Sedentary Elderly: A Randomised Controlled Trial

**DOI:** 10.1155/2019/3081029

**Published:** 2019-01-23

**Authors:** Tina-Thea Nielsen, Trine K. Møller, Lars L. Andersen, Mette K. Zebis, Peter R. Hansen, Peter Krustrup

**Affiliations:** ^1^Department of Nutrition, Exercise and Sports, University of Copenhagen, Copenhagen, Denmark; ^2^Department of Physiotherapy, University College Copenhagen, Copenhagen, Denmark; ^3^Department of Sports Science and Clinical Biomechanics, SDU Sport and Health Sciences Cluster (SHSC), Faculty of Health Sciences, University of Southern Denmark, Odense, Denmark; ^4^National Research Centre for the Working Environment, Copenhagen, Denmark; ^5^Department of Cardiology, Herlev and Gentofte University Hospital, Gentofte, Denmark; ^6^Sport and Health Sciences, University of Exeter, Exeter, UK

## Abstract

There is strong evidence that considerable health benefits can be achieved even with small amounts of physical activity. However, getting people to exercise regularly is a major challenge not least in the elderly population. This study investigated the feasibility and physiological health effects of a pragmatic 15-week exercise programme for sedentary elderly. In a single-blind randomised controlled trial, 45 sedentary 60-83-year-olds (25 women, 20 men) were randomly assigned (2:1 ratio) to a training group (TG, n=30) or a control group (CG, n=15). The training in TG consisted of a combination of exercise modalities (i.e., strength, aerobic fitness, stability, and flexibility training) performed once a week as supervised group-based training and a weekly home-based training for 15 weeks. Feasibility outcomes were exercise intensity, adherence, and adverse events. The primary outcome was change in aerobic fitness (VO_2max_/kg). Adherence was high (81%) for the supervised exercise and low (0%) for the home-based exercise. No acute injuries occurred in TG, but 4 subjects (13%) reported considerable joint pain related to training. Average heart rate (HR) during the supervised training was 104±12 beats/min (69.3±8.0%HR_max_), with 3.9±7.3% of training time >90%HR_max_. Intention-to-treat analyses revealed no between-group differences for aerobic fitness (P=0.790) or any secondary cardiovascular outcomes at 15-week follow-up (resting HR or blood pressure; P>0.05). Compared to CG, bodyweight (-2.3 kg, 95% CI -4.0 to -7.0; P=0.006), total fat mass (-2.0 kg, 95% CI -3.5 to -0.5; P=0.01), and total fat percentage (-1.6%, 95% CI -2.8 to -0.3; P=0.01) decreased in TG. The group-based supervised training had high adherence and moderate exercise intensity, whereas the home-based training was not feasible in this study population. This exercise programme performed once a week did not improve aerobic fitness. Thus, supervised training with more vigorous intensity control appears advisable. Clinical Study registration number is H-15016951.

## 1. Introduction

Physical inactivity is a major risk factor for noncommunicable diseases and premature death [1], and low cardiorespiratory fitness (CRF) is an independent risk factor for cardiovascular disease (CVD) and premature mortality [2]. Also, statutory retirement age is gradually increasing in most European countries, and keeping elderly people fit for work will be a major societal challenge. In Denmark, generations born in 1974 have to work until they are 70 years old, and retirement age will likely increase even more for future generations. Meta-analyses have provided strong evidence that exercise training significantly improves CRF and some CVD biomarkers in adults without CVD, which assigns an important role for exercise in the primary prevention of CVD [3]. Furthermore, there is strong evidence that exercise training is effective for prevention and treatment of noncommunicable metabolic and musculoskeletal disorders [4].

It is widely accepted that a dose-response relationship exists between exercise volume and possible health effects, but the minimum effective dose for eliciting positive health effects is unclear [5]. Addressing this issue is of crucial importance, because getting people to exercise regularly is a major challenge not least in the elderly population. A systematic review and a meta-analysis have revealed that considerable health benefits can be achieved even with small amounts of physical activity [6]. Indeed, one or two sessions per week of moderate- or vigorous-intensity leisure-time physical activity was associated with reduced all-cause mortality and death from CVD and cancer regardless of adherence to prevailing physical activity guidelines [6]. Moreover, 1 hour of recreational football per week over 12 weeks can produce health benefits by improving aerobic fitness (VO_2max_) and blood pressure in middle-aged sedentary men [7]. Research also indicates that women who did 1–1.5 hours of walking per week had only half the risk of developing coronary heart disease compared to sedentary women [8]. Furthermore, it has been suggested that a combination of aerobic training and strength training performed three times per week over 12 weeks can improve the aerobic fitness and muscle strength of elderly subjects [9]. However, it remains unknown whether a training programme consisting of a combination of exercise modalities (i.e., strength, aerobic fitness, stability, and flexibility training) performed twice a week provides a sufficient physiological stimulus to enhance the health of sedentary elderly.

In 2016, the Danish Gymnastics and Sports Associations (DGI) designed a combined exercise programme (combination of exercise modalities), namely, “DGI Senior training”, targeting elderly subjects with and without functional disability and noncommunicable diseases. The aim was to improve cardiovascular fitness and body composition (increase muscle mass and reduce fat mass) in the elderly population [10]. DGI hosts 6,300 local sports clubs across Denmark and by providing exercise programmes with health-enhancing effects, DGI could serve as a unique national platform with outreach also to the sedentary elderly population. However, to be able to provide evidence-based recommendations for health-enhancing exercise programmes for prevention of chronic diseases, the first step is to evaluate the effects of these programmes. Furthermore, to support the decision on whether an exercise programme is feasible on a population scale, this evaluation should be conducted in a real-life setting. High applicability makes it easier to extrapolate trial results into real life, which also makes such effectiveness studies highly relevant [11].

In the present study, the feasibility and physiological health effects of the “DGI Senior training” programme were evaluated in a randomised controlled trial. We hypothesised that this 15-week combined exercise programme could improve the health of sedentary elderly.

## 2. Methods

### 2.1. Experimental Approach to the Problem

This study was a randomised controlled trial that took place in the Capital Region of Denmark from February to June 2016. The study was conducted in a real-life setting in a local sports club hosted by DGI. Forty-five sedentary elderly men and women were randomised in a 2:1 ratio, to a training group (TG, n=30) or a matched control group (CG, n=15), in line with a prior intervention study in hypertensive patients [12]. After all baseline tests had been conducted, three numbers referring to three of the subjects, stratified by age, height, and sex, were placed in a sealed envelope. A person without any knowledge of the project performed the randomisation using following procedure with every sealed envelope: The first and second numbers drawn were placed in the training group and the third number drawn was placed in the control group. TG was offered the “DGI Senior training” programme which consists of 15 weeks of once a week supervised exercise training together with verbal advice and encouragement to also perform 30 min of exercise per week at home during this period as an integral part of the “DGI Senior training” concept. CG was advised to maintain their normal lifestyle behaviour during the intervention period and encouraged to contact the project manager if any changes in their normal activity and health occurred. The test personnel in charge of the VO_2max_ test after the 15-week intervention were blinded to group allocation and all baseline results. No other blinding procedure was possible due to financial constraints. The protocol was approved by the regional Ethical Committee (reference no. H-15016951). The guidelines of the Helsinki Declaration were followed and informed consent was obtained.

### 2.2. Subjects

A total of 45 sedentary elderly aged 60–83 years (20 men and 25 women) were included in the study. Baseline characteristics are presented in Tables 1 and 2. The subjects were recruited through advertisements in local newspapers and underwent a medical examination prior to inclusion in the study. Inclusion criteria were sedentary men and women above 60 years, who were healthy or with lifestyle diseases such as hypertension, chronic obstructive pulmonary disease, obesity, hyperlipidaemia, and/or CVD. The medical doctor responsible for the study safety predefined the following exclusion criteria: blood pressure >180/110 mmHg, polypharmacy (more than three types of prescribed medications), diabetes, ischaemic heart disease, blood donor status, implanted cardiac pacemaker, and moderate to severe dementia. The subjects were allowed to take prescribed medication and smoke during the intervention, but the medication was required to remain the same during the intervention period.

#### 2.2.1. Training Intervention

The intervention is described according to the Template for Intervention Description and Replication checklist and guide [13]. “DGI Senior training” consists of a 90 min supervised exercise programme once a week for 15 weeks conducted in a gym on a prescheduled weekday. Additionally, the subjects are encouraged to perform a 30-min home-based workout programme once a week, preferably 2–4 days after the supervised training. The home session should consist of two modalities from the training session held in the same training week (e.g., hip, ankle, pelvis, and chest/back flexibility, 2x30 sec balance standing on one leg and two strength exercises (squat to chair and chest press on chair), 12–15 reps, 2–3 sets).

The supervised training consisted first of a 12-min nonactive theory covering various health topics, followed by five training periods consisting of a 14-min warm-up period, a 16-min muscle endurance and strength training period, a 15-min moderate- to high-intensity training period, a 10-min core muscle stability period, and 10-min joint flexibility period. The supervised training sessions were provided in groups of 14-16 participants (both men and women) and mainly conducted as circuit training. Two female and one male instructor who had been given 16 hours of instruction in the training concept were in charge of the training.

Adjustable weight barrels and elastic bands were available for the supervised training. The progression of the muscle training began at a low intensity of 12–15 repetition maximum (RM) (muscle endurance training, sessions 1–5), followed by sessions 6–9 at a moderate intensity of 8–12 RM (strength training) and sessions 10–15 at a high intensity of 5–8 RM (strength training). The intensity progression was adjusted by the instructor in accordance with the subject's individual progression in both technique and strength.

The supervised training followed a weekly programme to standardise the delivery of the training. Adherence to the supervised training and home-based training was assessed by the instructors on a weekly basis. At the end of the study, the research project leader asked the participants if and how they had performed their home-based training. The self-reported adherence and training intensity were registered for each participant in a log book.

The medication used by the participants was registered by the medical doctor at the medical examination prior to the intervention and again at the postexamination. During the intervention, all participants were asked to report if any changes in medication and/or sickness occurred. No changes in medication occurred for any of the adherent subjects during the intervention (see Table 2). If a participant missed a session of supervised training, the project leader contacted them (1) to register the reason for absence and (2) to motivate them to keep attending the training sessions.

The role of the research group was to objectively describe and evaluate the programme without influencing the content, organisation, or intensity of the intervention. Data on characteristics of the supervised training sessions were collected. Four observations of total training sessions were conducted in intervention weeks 6, 8, 14, and 15. At the same training sessions, heart rates (HRs), accelerometer data, and rating of perceived exertion determined on a 0-10 visual analogue scale (VAS) were recorded in 29 subjects.

#### 2.2.2. Delivery of the Intervention

The supervised training consisted of five periods. The first period comprised 17 min (corresponding to 24% of total active training time) of varied, low-intensity, gymnastic warm-ups and walking followed by very light running. The gymnastic warm-ups were mainly arm swings and rotations for the whole body. Running and walking drills were often performed in pairs using a ball. The second period consisted of 10 min (14%) of cardiovascular training at low-to-moderate intensity, bouts of 10/20/30 s interval runs, relay races, stepping up and down on a step bench, interval runs 2 min with 1-min break, interval runs back and forth in the gym, running drills with a partner throwing a ball, or sweeping around the gym on rags. The third period consisted of 21 min (29%) of muscle endurance training consisting of a selection of seven exercises of 2–3 sets with 10-15 repetitions at 15–25 RM with adjustable barrels/elastics/body weight (squat, deadlift, bent over rowing, lunges, pull down, chest press, and shoulder press). The fourth period consisted of 12 min (17%) of core stability comprising static and dynamic whole-body exercises (2x30 sec to exhaustion). The fifth period consisted of 10 min (14%) of flexibility/cool-down.

### 2.3. Outcomes

The primary outcome was aerobic fitness calculated as VO_2max_ expressed relative to bodyweight.

The secondary outcomes were body composition variables including lean body mass, bone mass, bone density, and whole-body and regional fat mass. Furthermore, an exploratory analysis was performed to investigate the potential between-group effects in supplementary health outcome variables: (1) resting HR and blood pressure, (2) metabolic health variables including fasting blood lipids, high sensitive C-reactive protein (hsCRP), insulin, and HbA1c, (3) bone health variables including concentrations of plasma bone turnover markers (BTMs) (*µ*g/l), i.e., procollagen type 1 amino-terminal propeptide (P1NP), osteocalcin (Oc), sclerostin, and C-terminal telopeptide of type 1 collagen (CTX-1).

### 2.4. Procedures

The study protocol investigated the cardiovascular and musculoskeletal adaptations as well as the health status of the subjects. All subjects were tested before and after the 15-week intervention at the same time of the day (7–11 am). They were told to attend fasting with no medicine intake or smoking since the preceding midnight.

#### 2.4.1. Aerobic Fitness

VO_2max_ was determined by pulmonary gas exchange measurements (Master Screen CPX, Viasys Healthcare, St Paul, Minnesota, USA) starting at 40 W for a 2-min period followed by a 20-W increase every 2 min until exhaustion. To objectively confirm achievement of VO_2max_, a levelling off in oxygen uptake and/or RER>1.05 was used. VO_2_ was determined as mean values over 30 s and VO_2max_ defined as the highest 30 s mean value.

#### 2.4.2. Body Composition

Lean body mass, bone mass, bone density, and whole body and regional fat mass were determined by dual-energy X-ray absorptiometry (DEXA scan; LUNAR, GE Medical Systems, Madison, Wisconsin, USA).

#### 2.4.3. Blood Samples

After a medical examination, blood samples were drawn for immediate testing of blood glucose, lipids, insulin, glycated haemoglobin (HbA1c), and hsCRP according to standardised laboratory procedures. 4 ml of the blood was centrifuged immediately and serum separated and stored in small microcentrifuge tubes for 24 hours at -20°C and subsequently at -80°C until further analysis. The concentration of plasma BTMs was assessed by a chemiluminescence method using a fully automated immunoassay system (iSYS, Immunodiagnostic Systems Ltd., Boldon, England). The intermediary precision coefficient of variation ranged from 8 to 10%. Analyses were performed at the Department of Clinical Biochemistry, Rigshospitalet, Glostrup, Denmark. Prior to the DXA scan, body height and weight were measured.

#### 2.4.4. Blood Pressure

After at least 10 min of rest in a dark, temperate room, lying in a supine position, the resting blood pressure was assessed using an automatic upper left arm blood pressure monitor (OMRON-M7; OMRON; Illinois, USA). Six measurements of systolic (SBP) and diastolic blood pressure (DBP) were made and the mean arterial blood pressure (MAP) was calculated. Simultaneous resting HR was measured with Polar Team System, Polar Electro Oy and determined as the lowest average value over 1 min.

#### 2.4.5. Heart Rate Responses

HR was recorded every 5 sec using a telemetric device (Polar Team System, Polar Oy, Kempele, Finland). The variables used were percentage of time spent in each intensity zone in percent of maximum HR (%HR_max_) and relative values in relation to the mean HR (%HR_mean_). HR_max_ was obtained during the incremental VO_2max_ test or during training. In a few cases, higher HR_max_ was measured during training sessions than during the bicycle test. In all cases the highest achieved heart rate was used as HR_max_.

#### 2.4.6. Musculoskeletal Impact: Player Load Measurements

To determine the musculoskeletal impact, player load (PL) was obtained via accelerometry, combining the accelerations produced in three planes of body movement by means of a 100-Hz triaxial accelerometer. Accumulated PL (*r*) is an estimate of physical demand combining the instantaneous rate of change in acceleration in three planes, namely, forward/backward X, sideways Y, and up/down Z, using the following formula presented in the following equation:

(1) Accumulated  player  loadr=∑t=0t=nXt=n−Xt=n−12+Yt=n−Yt=n−12+Zt=n−Zt=n−12

The validity and reliability of the GPS units and the incorporated accelerometers have been described elsewhere [14]. By measuring PL, total PL, PL/min, and average time spent in PL zones 0–1, 1–2, 2–3, 4–6, total accelerations, average number of jumps, and average percentage of PL in three different planes (forward, sideways, and upwards) were detected. The accelerations were summarised as low (1.50–2.14 m·s^−2^), moderate (2.14–2.78 m·s^−2^), and high (>2.78 m·s^−2^).

### 2.5. Statistical Analyses

Analyses were performed using SAS statistical software (SAS version 9.4). The changes from baseline to follow-up between the two groups were evaluated using a linear mixed model. The change score was adjusted for the baseline value of the outcome, age, and gender. The estimation method was restricted maximum likelihood with degrees of freedom based on the Satterthwaite approximation. P levels of 0.05 or less were accepted as statistically significant. Outcomes are reported as within- and between-group least square mean differences with 95% confidence intervals of the change score from baseline to 10-week follow-up.

#### 2.5.1. Sample Size

The a priori power calculation was based on aerobic fitness (expressed relative to body mass) as the primary outcome. To show an expected increase of 6% in VO_2max_ would require 15 participants in the control group and 30 participants in the training group to detect between-group significance at a level of 0.05%, according to the 2:1 randomisation. The expected increase in VO_2max_ was based on an expected drop out of participants of 20% [12, 15]. No power calculations were carried out for the remainder of the variables.

## 3. Results

### 3.1. Baseline Data

**Figure 1:**
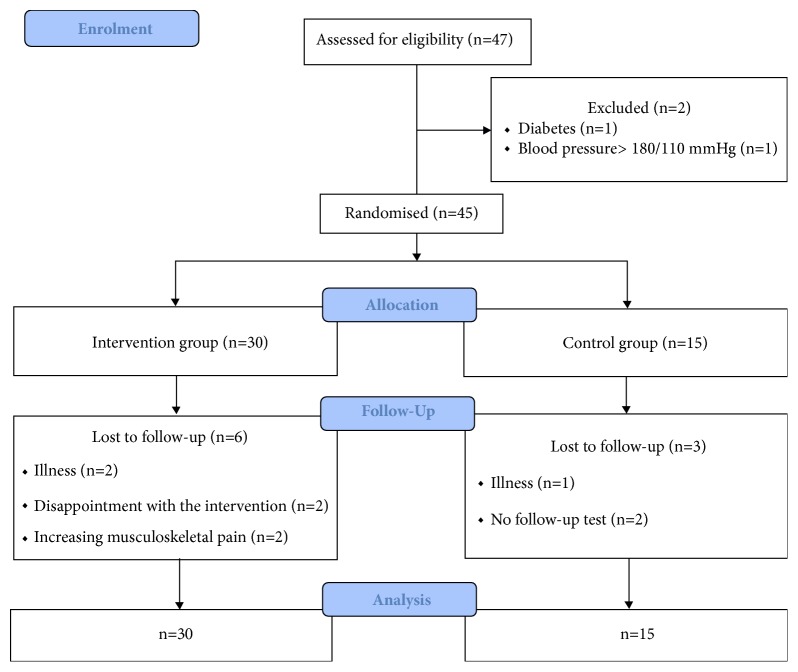
Study flow chart.

No baseline differences between TG and CG were observed for aerobic fitness, body composition, or blood pressure; see Table 1. Likewise, no baseline differences were observed for blood lipids, HbA1c, or BTMs; see Table 2. Participant flow is presented in Figure 1, and baseline characteristics for the participants included in the intention-to-treat analysis are presented in Tables 1 and 2.

### 3.2. Feasibility

The total number of supervised training sessions was 15, corresponding to one session per week over the 15-week intervention period. The attendance rate was 81%. The attendance rate for the home-based training was 0%. The main reasons for the participants not performing the home-based training were (1) lack of motivation to train on their own and (2) uncertainty about what drills to perform and how to perform them. Total average training time was 72.9±4.3 min. Mean HR during training was 104±12 beats/min, corresponding to 69.3±8.0% of individual HR_max_.

### 3.3. Training Intensity

**Figure 2:**
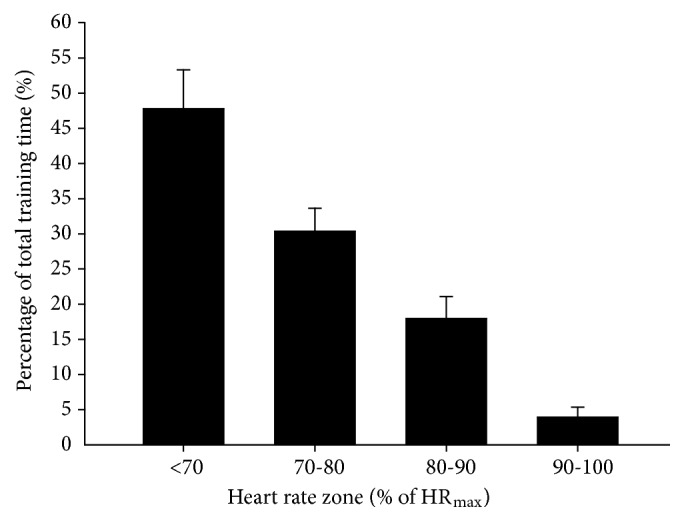
Heart rate distribution during exercise, expressed in percentage of total training time in selected heart rate zones. Data are presented as mean ± SEM.

Average percentage of total training time spent in HR zones <70%, 70–80%, and 80–90% was 47.8±28.3, 30.4±16.7, and 18.0±15.9%, respectively (Figure 2). The fraction of training time spent at >80% of HR_max_ was 21.9±21.7% of total training time, while 3.9±7.3% of training time was spent at >90%  HR_max_ (n=26). Three of the subjects never reached an intensity >80% of HR_max_, and seven of the subjects never reached an intensity >90% of HR_max_. On average, total PL was 121±37 arbitrary units, corresponding to 2±0 PL/min. The average fraction of time spent in PL zone 0–1 was 98±1%, while the average fraction of time spent in PL zones 1–2, 2–3, and 4–6 was in each case 0±0. The total numbers of jumps were 3±3, while the total numbers of low accelerations, moderate accelerations, and high accelerations were 7±4, 3±2, and 3±3, respectively. The fraction of time with player load in the forward plane (1D Fwd), sideways plane (1D Side), and upwards plane (1D UP) was 27±1, 31±2, and 41±2%, respectively. To test for a dose-response relationship between PL and gain in muscle mass, we divided TG into three subgroups: lowest PL (n=8), moderate PL (n=9), and highest PL (n=9). The subjects reported overall RPE values of 6.1±1.7 on a 0–10 VAS. Specified RPE values for the legs and for the respiration were 5.2±2.0 and 4.8±2.3, respectively (n=26).

### 3.4. Adverse Events

Two participants reported joint pain related to the training and therefore decided to drop out of the study. Two participants experienced severe knee joint pain at the end of the intervention period. No acute injuries occurred during training or in the control group.

### 3.5. Primary Outcome

**Figure 3:**
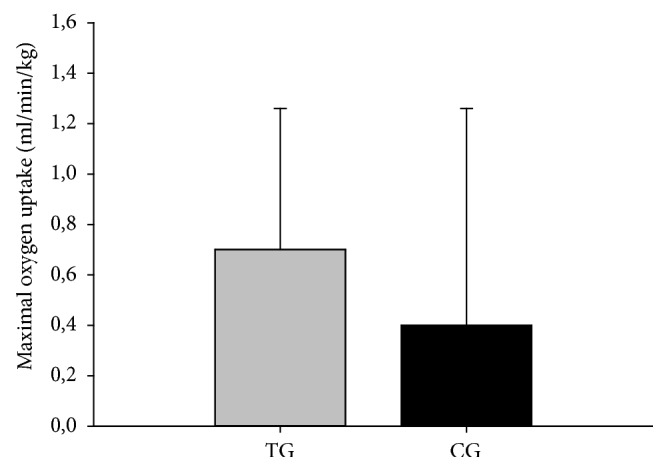
Maximal oxygen uptake expressed in relation to body weight. Changes scores presented as mean ± SEM for TG versus CG after 15 weeks (n=45).

No between-group effects were observed for VO_2max_ (P=0.423) or VO_2max_/kg (P=0.790); see Table 3. After the 15-week intervention, VO_2max_ was unaltered in both TG (15.5 mLO_2_/min, 95% CI -75.3 to 106.4, P=0.729) and CG (84.9 mLO_2_/min, 95% CI -58.8 to 228.7, P=0.236). VO_2max_ expressed in relation to body mass was unaltered in both TG (0.7 mLO_2_/min/kg, 95% CI -0.4 to 1.8, P=0.205) and CG (0.4 mLO_2_/min/kg, 95% CI -1.3 to 2.1, P=0.603); see Figure 3.

### 3.6. Secondary Outcomes

#### 3.6.1. Body Composition

**Figure 4:**
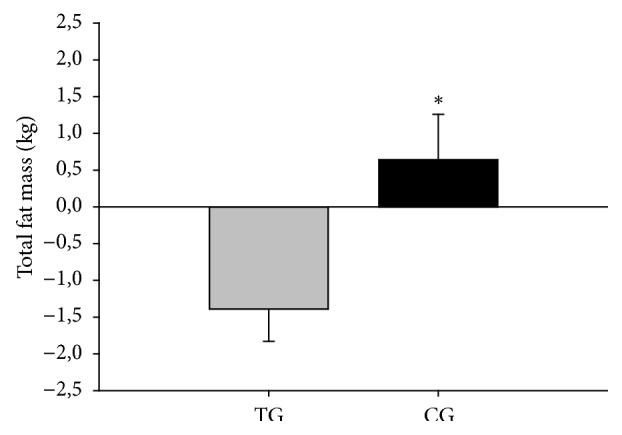
Total fat mass (kg). Change scores presented as mean ± SEM. *∗* denotes the significant difference of TG versus CG after 15 weeks (*P<0.05*) (n=45).

After 15 weeks, a between-group effect in favour of TG was observed for total body mass (P=0.006, effect size [ES] 0.2), BMI (P=0.004; ES 0.2), whole-body fat percentage (P=0.014; ES 0.2), total fat mass (kg) (P=0.010; ES 0.2) (see Figure 4), android fat mass (P=0.010; ES 0.2), and gynoid fat mass (P=0.003; ES 0.2). We observed no between-group effect on muscle mass (P=0.974), bone mineral density (P=0.222), or bone mineral content (P=0.712); see Table 3.

Total body mass was lowered in TG (-1.1 kg, 95% CI -2.0 to -0.2, P=0.020), while tending to rise in CG (1.3 kg, 95% CI -0.0 to 2.6, P=0.058). BMI was reduced in TG (-0.5 kg/m^2^, 95% CI -0.8 to -0.1, P=0.005), while tending to be increased in CG (0.4 kg/m^2^, 95% CI -0.1 to 0.8, P=0.092). Total fat percentage was reduced in TG (-1.4 %, 95% CI -2.1 to -0.7, P<0.001) and unaltered in CG (0.2 %, 95% CI -0.8 to 1.2, P=0.724). Moreover, total fat mass was lowered in TG (-1.4 kg, 95% CI -2.2 to -0.5, P=0.002) and unaltered in CG (0.6 kg, 95% CI -0.6 to 1.9, P=0.292). Android fat mass was also lowered in TG (-0.2 kg, 95% CI -0.3 to -0.1, P<0.001) and unaltered in CG (0.1 kg, 95% CI -0.1 to 0.2, P=0.335). Likewise, gynoid fat mass was lowered in TG (-0.3 kg, 95% CI -0.4 to -0.2, P<0.001) and unaltered in CG (0.0 kg, 95% CI -0.2 to 0.3, P= 0.646). Total muscle mass tended to slightly increase in TG (0.4 kg, 95% CI 0.0 to 0.9, P=0.062) but was unaltered in CG (0.5 kg, 95% CI -0.2 to 1.2, P=0.181). Bone mineral density was unaltered for the TG (0.000 g/cm^2^, 95% CI -0.001 to 0.011, P=0.123) but improved in CG (0.012 g/cm^2^, 95% CI 0.003 to 0.021, P=0.012). Bone mineral content showed a reduction in TG (-0.004 kg, 95% CI -0.068 to -0.012, P=0.007) but was unchanged in CG (-0.030 kg, 95% CI -0.071 to 0.010, P=0.137).

#### 3.6.2. Cardiovascular Health Variables

No between-group effects were observed in resting HR (P=0.895), SBP (P=0.752), DBP (P=0.522), or MAP (P=0.597); see Table 3. After the 15-week intervention, mean resting HR was unaltered in TG (1 bpm, 95% CI -1 to 3, P=0.193) and in CG (1 bpm, 95% CI -2 to 4, P=0.446). In TG, there was a reduction in SBP (-5 mmHg, 95% CI -9 to -1, P=0.028), but CG was unaltered (-4 mmHg, 95% CI -10 to 2, P=0.228). DBP was lowered in TG (-4 mmHg, 95% CI -6 to -1, P=0.003) but unaltered in CG (-2 mmHg, 95% CI -5 to 1, P=0.155), while MAP was reduced in TG (-4 mmHg, 95% CI -7 to -1, P=0.006) and unaltered in CG (-3 mmHg, 95% CI -7 to 1, P=0.169).

#### 3.6.3. Metabolic Health Variables

No between-group effects were observed for levels of total cholesterol (P=0.254), high density lipoprotein (HDL)-cholesterol (P=0.165), low density lipoprotein (LDL)-cholesterol (P=0.264), triglyceride (P=0.919), total/HDL cholesterol ratio (P=0.404), fasting insulin (P=0.451), HbA1c% (P=0.810), hsCRP (P=0.794), and eAG (P=0.669); see Table 3. A reduction in fasting insulin levels was observed in TG (-27.7 pmol/L, 95% CI -38.4 to -17.0, P<0.0001), but also in CG (-34.5 pmol/L, 95% CI -49.1 to -19.9, P<0.001). HbA1c% was increased in TG (0.1 %, 95% CI 0.0 to 0.1, P=0.012) and unaltered in CG (0.0 %, 95% CI 0.0 to 0.1, P=0.115). Plasma triglycerides were lowered in TG (-0.2 mmol/L, 95% CI -0.3 to 0.0, P=0.020) but were not significantly altered in CG (-0.2 mmol/L, 95% CI -0.4 to 0.0, P=0.071).

#### 3.6.4. Bone Health Variables

No between-group effects were observed for concentrations of osteocalcin (P=0.379), CTX-1 (P=0.705), or sclerostin (P=0.696), while a tendency towards a difference in P1NP was observed (P=0.066); see Table 3. P1NP showed an increase in TG (11.5 *µ*g/L, 95% CI 5.1 to 18.0, P=0.001) while P1NP was unaltered in CG (1.4 *µ*g/L, 95% CI -7.2 to 10.0, P=0.744). There were no other within-group effects for the bone markers.

## 4. Discussion

The present study evaluated the feasibility and physiological effects of the “DGI Senior training” programme. The supervised training was conducted at moderate aerobic and musculoskeletal intensities. While adherence to the supervised exercise training was high, adherence to home-based training was nil. “DGI Senior training” performed as one session per week over 15 weeks did not change the primary outcome of aerobic fitness. However, there were significant reductions in bodyweight, BMI, and total fat percentage in TG at follow-up, while the lean body mass, bone mineralisation, and BTMs were unaltered. There were no significant improvements in blood pressure, resting HR, blood lipid profile, or HbA1c levels.

### 4.1. Feasibility of “DGI Senior Training”

The supervised component of “DGI Senior training” is a mix of exercise modalities of mainly aerobic, muscle endurance, stability, and flexibility training carried out once a week at moderate intensity and is therefore regarded as a low-frequency, moderate-intensity activity. The training was organised as a stationary circuit workout, with only brief bouts of specific cardiovascular drills with high intensity.

The musculoskeletal impact in this study was lower compared to other interventions showing health improvements in untrained men [16] and recreationally active young [17], and no correlations were found between PL and adherence to training. An ambition of the “DGI Senior training” was to improve muscle mass through strength training by both supervised training and home-based training, respectively, giving a training frequency of two times per week. However, adherence to home-based training was nil and a lower than expected intensity during the supervised muscle training sequences was observed. The execution of the strength training modality in the present study is representative of muscle endurance training (not muscle strength training) which is considered insufficient to increase the muscle mass. Apparently, it was not possible to achieve progressive muscle training during the study, probably because the group was very heterogeneous in relation to gaining experience with training with barrels which restricted the technical and PL level of each exercise. Indeed, the subjects rated their perceived exertion level as moderate, suggesting they could have been challenged to achieve higher training intensities. Furthermore, the participants lacked motivation to perform the home-based training. They felt uncertain about what drills to perform and how to do so. This suggests that the delivery of home-based exercise in the “DGI Senior training” programme is not feasible in this population.

### 4.2. Physiological Effects

#### 4.2.1. Primary Study Outcome

It is relevant to evaluate the fitness and health effects of training according to the model by Krustrup et al. 2018 [18], separating the training effects into subcategories of cardiovascular fitness, metabolic fitness, and skeletal fitness. With regard to aerobic fitness, no improvements were observed for either VO_2max_ or VO_2max_ relative to body mass despite a decrease in total body weight. This is somewhat surprising given the sedentary status of the study subjects and their low level of aerobic fitness at baseline. High-intensity aerobic training has a significant impact on aerobic fitness and seems to be superior to continuous moderate-intensity exercise for improving aerobic fitness and cardiovascular health [19, 20]. Indeed, training comprising high-intensity activity twice a week has shown broad spectrum health effects in untrained elderly men and women [21, 22], and a high-intensity activity like football has been shown to improve VO_2max_ by 16% over 16 weeks for 65–75-year-old untrained men subjected to 2 times 60 min of training per week [23]. However, low-frequency, moderate-intensity training like walking football once a week did not improve aerobic fitness in elderly subjects [24], while a walking football study with twice the training volume showed significant improvements in SBP, fat mass, and exercise tolerance, but only a nonsignificant 5% improvement in VO_2peak_ relative to bodyweight [25]. Our study revealed that one weekly training session over 15 weeks, with an average HR of 69% of HRmax, and less than 4% of training time with HRs in the highest intensity zone above 90% of HRmax was not sufficient to elicit significant effects in aerobic fitness.

#### 4.2.2. Secondary Study Outcomes

At baseline, total fat percentage was high in both groups which is believed to constitute a risk factor for CVD [26, 27]. The reduction in total fat mass of 1.4 kg after 15 weeks of training observed in the present study is similar to results from previous studies involving elderly subjects training at low-frequency, moderate-intensity [25, 28]. We detected a fat loss of 0.2 kg in the android region and 0.3 kg in the gynoid region, and it has been demonstrated that a reduction in visceral fat mass can be achieved by either caloric restriction or aerobic exercise with a frequency as low as 1–2 times per week [29]. This reduction in android fat mass is considered to be positive for metabolic health, as excess body fat, particularly abdominal fat, is associated with insulin resistance, hyperlipidaemia, and increased risk of CVD [30].

PL data from our study suggest that the musculoskeletal impact was of low-frequency, low-to-moderate intensity which is insufficient to induce significant muscle hypertrophy. Other low-frequency activities like small-sided street soccer [17] and small-sided basketball [16] have induced gains in muscle mass, but these activities involve a higher number of accelerations and PL/min compared to the current “DGI Senior training” data. To improve bone health, multidirectional dynamic loading that induces relatively high bone strain at high speed is required [31]. In our study, the training intervention had a low dynamic load which may explain why we did not find improvements in bone health parameters. Positive effects on other important cardiovascular risk factors, e.g., BP, resting HR, peak ventilation, and time to exhaustion, were not observed after 15 weeks of low-frequency, moderate-intensity training.

In summary, the only positive health effect was on metabolic fitness (i.e., weight loss). It is worth mentioning that according to recommendations on physical activity [4] the volume and the intensity of the present intervention were insufficient which may explain why the intervention did not result in any other health improvements.

## 5. Conclusion

This pragmatic “DGI Senior training” 15-week exercise programme elicited moderate aerobic and musculoskeletal exercise intensities in sedentary elderly subjects. In this setting, only the supervised (not the home-based) training was feasible. The training programme elicited no improvements in aerobic fitness, probably because of the low exercise volume conducted at moderate aerobic intensity. Likewise, no effects were found in musculoskeletal fitness, whereas limited positive effects were observed in body composition suggestive of improved metabolic fitness. These results indicate limited health effects of the “DGI Senior training” programme and an increase in supervised training frequency and intensity may be advisable.

## 6. Strengths and Limitations

A strength of the study is that this was a randomised controlled trial which eliminates most types of bias associated with nonrandomised studies. For the exploratory analysis, we did not have sufficient power to draw firm conclusions about any of the variables included in our analyses. There was no considerable familiarisation with the training programme for study subjects prior to the intervention, which probably contributed to failure to achieve the intended intensity during the muscle training sessions. Finally, we did not formally register the participant's diet which may have affected the results.

## 7. Practical Applications

The 15-week “DGI Senior training” exercise programme was designed to include several training modalities, i.e., strength, aerobic fitness, stability, and flexibility training, in order to improve aerobic fitness and body composition. This study revealed that combining these various training modalities in a 70 min training session resulted in no improvement in aerobic fitness and minor improvements in body composition. We, therefore, recommend focusing on cardiovascular training and muscle strength training with vigorous intensity control. RPE showed a moderate intensity similar to the heart rate measurements. Therefore, it is suggested to apply the use of RPE as a tool for the instructors to monitor the intensity of the participants during each training session. During the cardiovascular training, it is recommended to consider more playful activities/games of moderate to high intensity. To ensure a safe progression of the strength training, prior habituation is needed and the specific drills must be individually differentiated and adjusted according to the technical level of each participant. Finally, individually organised home-based training is not feasible in this population group. Preferably the home-based training should be converted to supervised training or as a minimum be described with progression on a weekly basis and arranged in groups.

## Figures and Tables

**Table 1:** Subject characteristics at baseline.

	**TG (n = 30)**	**CG (n = 15)**	**Between-group**
	**baseline**	**baseline**	***P-value***
Men/women (n)	13/17	7/8	
Age (years)	71 ± 6	70 ± 6	0.620
Weight (kg)	77.6 ± 17.0	81.8 ± 10.3	0.307
Height (cm)	168.0 ± 8.7	173.4 ± 8.3	0.050
Body Mass Index (kg/m^2^)	27.4 ± 5.2	27.3 ± 3.4	0.931
Total fat percentage	34.5 ± 8.8	35.9 ± 9.7	0.628
Total fat mass (kg)	27.6 ± 11.2	29.6 ± 10.1	0.549
Android fat mass (kg)	2.7 ± 1.3	2.9 ± 1.0	0.715
Gynoid fat mass (kg)	4.7 ± 1.8	5.1 ± 2.0	0.585
Total muscle mass (kg)	46.4 ± 9.0	48.3 ± 9.0	0.503
Leg muscle mass (kg)	15.5 ± 3.3	16.4 ± 3.6	0.424
Total bone mineral density (g/cm^2^)	1.149 ± 0.154	1.187 ± 0.112	0.406
Total bone mineral content (kg)	2.643 ± 0.707	2.919 ± 0.615	0.205

Resting heart rate (bpm)	62 ± 9	60 ± 7	0.647
Systolic blood pressure (mmHg)	137 ± 15	136 ± 17	0.901
Diastolic blood pressure (mmHg)	77 ± 9	79 ± 7	0.417
Mean arterial blood pressure (mmHg)	97 ± 10	98 ± 9	0.692

VO_2max_ (mLO_2_/min)	1598 ± 76	1739 ± 24	0.380
VO_2max_ (mLO_2_/min/kg)	20.7 ± 4.7	21.3 ± 5.7	0.718
VEpeak (L/min)	70.6 ± 25.0	74.4 ± 15.0	0.628
Time to exhaustion (s)	384 ± 107	421 ± 116	0.307

Data are presented as mean ± SD. TG: training group. CG: control group. *∗* denotes significant difference between TG and CG *(P<0.05)*.

**Table 2:** Subject characteristics at baseline.

	**TG (n = 30)**	**CG (n = 15)**	**Between-group**
	**Baseline**	**Baseline**	*** P-value***
CRP (mg/L)	3.6 ± 3.3	3.1 ± 3.1	0.606
eAG (mmol/L)	6.5 ± 0.4	6.5 ± 0.4	0.646
HbA1c %	5.7 ± 0.3	5.7 ± 0.2	0.761
Fasting insulin (pmol/L)	83.1 ± 45.1	79.8 ± 31.3	0.799
Total Cholesterol (mmol/L)	5.9 ± 0.8	5.4 ± 0.7	0.069
HDL-Cholesterol (mmol/L)	1.7 ± 0.5	1.8 ± 0.3	0.754
LDL-Cholesterol (mmol/L)	3.6 ± 0.8	3.2 ± 0.5	0.147
Triglycerides (mmol/L)	1.5 ± 0.9	1.2 ± 0.4	0.130
Total Chol/HDL ratio	3.69 ± 1.15	3.16 ± 0.57	** 0.047** **∗**
Osteocalcin (*µ*g/L)	20.4 ± 11.5	21.3 ± 7.7	0.791
P1NP (*µ*g/L)	52.5 ± 23.2	57.7 ± 18.2	0.453
CTX-1 (*µ*g/L)	0.4 ± 0.2	0.5 ± 0.3	0.145
Sclerostin (ng/mL)	0.9 ± 0.4	0.8 ± 0.2	0.141
**Medical status**			
On/off medication (n)	18/12	10/5	
On/off medication (%)	60/40	67/33	
Healthy (%)	20	20	
**Lifestyle diseases**			
Asthma (%)	3	7	
COL (%)	3	0	
Hypertension (%)	20	47	
Hypercholesterolemia (%)	7	20	
**Musculoskeletal diseases**			
Osteoporosis (%)	20	0	
OA (%)	30	13	
Herniated disc (%)	3	7	
**Other diseases (**%**)**	40	27	

Data are presented as mean ± SD. TG: training group. CG: control group. *∗* denotes significant difference between TG and CG *(P<0.05). *

**Table 3:** Between-group difference after 15 weeks of training.

	**TG**	**(n=30)**	**CG**	**(n=15)**	**Between-Group**		
					**Difference**		
	**Change**	**(95**%** CI)**	**Change**	**(95**%** CI)**		**(95**%** CI)**	*** P- Value***
**Body composition**							
Weight (kg)	-1.1	(-2.0 to -0.2)	1.3	(-0.0 to 2.6)	-2.3*∗∗*	(-4.0 to -0.7)	**0.006**
Body Mass Index (kg/m^2^)	-0.5	(-0.8 to -0.1)	0.4	(-0.1 to 0.8)	-0.8*∗∗*	(-1.4 to -0.3)	**0.004**
Total muscle mass (kg)	0.4	(0.0 to 0.9)	0.5	(-0.2 to 1.2)	0.0	(-0.9 to 0.9)	0.974
Total fat percentage (%)	-1.4	(-2.1 to -0.7)	0.2	(-0.8 to 1.2)	-1.6*∗*	(-2.8 to -0.3)	**0.014**
Total fat mass (kg)	-1.4	(-2.2 to -0.5)	0.6	(-0.6 to 1.9)	-2.0*∗*	(-3.5 to -0.5)	**0.010**
Android fat mass (kg)	-0.2	(-0.3 to -0.1)	0.1	(-0.1 to 0.2)	-0.3*∗∗*	(-0.4 to -0.1)	**0.003**
Gynoid fat mass (kg)	-0.3	(-0.4 to -0.2)	0.0	(-0.2 to 0.3)	-0.3*∗*	(-0.6 to -0.1)	**0.010**
Total bone mineral density (g/cm^2^)	0.000	(-0.001 to 0.011)	0.012	(0.003 to 0.021)	-0.007	(-0.018 to 0.004)	0.220
Total bone mineral content (kg)	-0.004	(-0.068 to -0.012)	-0.030	(-0.071 to 0.010)	-0.009	(-0.060 to 0.042)	0.712
**Aerobic Fitness**							
VO_2max_ (mLO_2_/min)	15.5	(-75.3 to 106.4)	84.9	(-58.8 to 228.7)	-69.4	(-244.3 to 105.5)	0.423
VO_2max_ (mL/min/kg)	0.7	(-0.4 to 1.8)	0.4	(-1.3 to 2.1)	0.3	(-1.8 to 2.3)	0.790
Resting heart rate (bpm)	1	(-1 to 3)	1	(-2 to 4)	0	(-3 to 4)	0.895
**Blood pressure**							
Systolic blood pressure (mmHg)	-5	(-9 to -1)	-4	(-10 to 2)	-1	(-9 to 7)	0.752
Diastolic blood pressure (mmHg)	-4	(-6 to -1)	-2	(-5 to 1)	-1	(-5 to 3)	0.522
Mean arterial blood pressure (mmHg)	-4	(-7 to -1)	-3	(-7 to 1)	-1	(-6 to 4)	0.597
**Lipids**							
Total Cholesterol (mmol/L)	-0.2	(-0.4 to 0.1)	0.1	(-0.3 to 0.5)	-0.3	(-0.7 to 0.2)	0.254
HDL-Cholesterol (mmol/L)	0.0	(-0.1 to 0.1)	0.1	(0.0 to 0.2)	-0.1	(-0.2 to 0.0)	0.165
LDL-Cholesterol (mmol/L)	0.0	(-0.2 to 0.3)	0.3	(0.0 to 0.6)	-0.2	(-0.6 to 0.2)	0.264
Triglyceride (mmol/L)	-0.2	(-0.3 to 0.0)	-0.2	(-0.4 to 0.0)	0.0	(-0.3 to 0.3)	0.919
Total Chol/HDL ratio	-0.2	(-0.4 to 0.1)	0.0	(-0.3 to 0.3	-0.2	(-0.6 to 0.2)	0.404
**Glycemic control**							
Fasting insulin (pmol/L)	-27.7	(-38.4 to -17.0)	-34.5	(-49.1 to -19.9)	6.8	(-11.4 to 25.0)	0.451
HbA1c %	0.1	(0.0 to 0.1)	0.0	(0.0 to 0.1)	0.0	(-0.1 to 0.1)	0.810

CRP (mg/L)	-0.4	(-1.2 to 0.3)	-0.6	(-1.6 to 0.4)	0.2	(-1.1 to 1.4)	0.794
eAG (mmol/L)	0.1	(0.0 to 0.2)	0.1	(0.0 to 0.2)	0.0	(-0.1 to 0.2)	0.669
**Bone markers**							
Osteocalcin (*µ*g/L)	1.5	(-0.4 to 3.5)	0.1	(-2.5 to 2.7)	1.4	(-1.8 to 4.7)	0.379
P1NP (*µ*g/L)	11.5	(5.1 to 18.0)	1.4	(-7.2 to 10.0)	10.1	(-0.7 to 20.9)	0.066
CTX-1 (*µ*g/L)	-0.0	(-0.1 to 0.1)	0.0	(-0.1 to 0.1)	0.0	(-0.1 to 0.1)	0.705
Sclerostin (ng/mL)	0.05	(-0.0 to 0.1)	0.03	(-0.1 to 0.1)	0.02	(-0.1 to 0.1)	0.696

TG: training group. CG: control group. Within-group data are presented as mean change (95% CI) and between-group data as estimated mean difference (95% CI). *∗* denotes significant between-group effect *(P<0.05)*  *∗∗*  *(P<0.01).*

## Data Availability

The data used to support the findings of this study are available from the corresponding author upon request.
